# The Effect of Endoscopic Olfactory Cleft Opening on Obstructed Olfactory Cleft Disease

**DOI:** 10.1155/2020/8073726

**Published:** 2020-03-27

**Authors:** Rong-San Jiang, Kai-Li Liang

**Affiliations:** ^1^Departments of Medical Research, Taichung Veterans General Hospital, Taichung, Taiwan; ^2^Departments of Otolaryngology, Taichung Veterans General Hospital, Taichung, Taiwan; ^3^School of Medicine, Chung Shan Medical University, Taichung, Taiwan; ^4^Rong Hsing Research Center For Translational Medicine, National Chung Hsing University, Taichung, Taiwan; ^5^Faculty of Medicine, National Yang-Ming Medical University, Taipei, Taiwan

## Abstract

**Purpose:**

This study was conducted to evaluate the effect of endoscopic olfactory cleft (OC) opening on olfaction in patients with obstructed OC disease. *Materials and Methods*. Patients with obstructed OC disease who underwent endoscopic OC opening for treatment were enrolled. The endoscopic olfactory cleft opening was performed under local anesthesia. Under an endoscopy, the middle and superior turbinates were gently lateralized to open the OC using an elevator. The phenyl ethyl alcohol threshold test was performed to evaluate the olfactory function both before and after surgery.

**Results:**

An endoscopic OC opening was performed on 42 patients. Amongst them, the etiology of OC obstruction revealed anatomic anomalies in 14 patients, inflammatory process in 14, and anatomic anomalies as well as inflammatory process in 14. The phenyl ethyl alcohol threshold levels improved in 32 (76.2%) of the patients after surgery. The olfactory function was better improved in patients experiencing OC obstructed by inflammatory process than those by anatomic anomalies.

**Conclusions:**

This study showed that endoscopic OC opening seemed to be effective in treating olfactory dysfunction in patients with obstructed OC disease caused by inflammatory process.

## 1. Introduction

Free access of air to the olfactory cleft (OC) is a key element towards maintaining normal olfactory function [1]. Biacabe et al. have defined OC disease as an olfactory disability related with a clinical and/or radiologic OC abnormality, involving a pathologic process limited to or predominating in the OC [2]. They classified the OC disease into 3 groups based on the etiology. The first group was anosmia with OC malformation. The second group was the OC obstructed by anatomic deformities associated with an inflammatory process. The third group was the OC obstructed only by an inflammatory process.

Corticosteroids are the most frequently prescribed drugs used to treat olfactory dysfunction [3]. An intranasal corticosteroid application produces fewer side effects than do oral corticosteroids [4]. However, a conventional intranasal corticosteroid application has been considered as having little effect on olfactory dysfunction due to the inability of topical corticosteroids to reach the OC [3, 5]. Therefore, several modified modalities of delivery have been recommended in order to help improve the access of topical corticosteroids to the OC [3–5]. When the OC is obstructed by a local inflammation of mucosa with a stagnation of secretion, the olfactory function is often nonresponsive to medical treatment [1]. Only a 25% improvement rate in olfactory thresholds was achieved in those with OC disease after medical therapy with an oral corticosteroid (prednisolone 1 mg/kg weight/day) for 6 days, along with an intranasal corticosteroid for 1 month [2].

Endoscopic OC surgery has been used to treat diseases related to the OC [6]. Endoscopic removal of polyps from the OC has been shown to improve olfactory function [7]. Recently, OC dilatation has been reported to treat constitutional OC stenosis in 3 cases. Improvement in dysosmia was achieved after surgery [8]. The aim of the study was to evaluate the effect of endoscopic OC opening on OC disease obstructed by anatomic deformities and/or an inflammatory process.

## 2. Materials and Methods

### 2.1. Subjects

**Figure 1:**
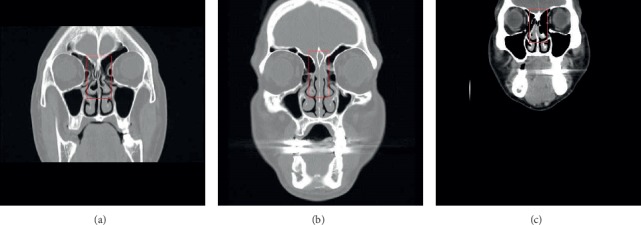
(a) Obstructed olfactory cleft caused by anatomic anomalies. (b) Obstructed olfactory cleft caused by the inflammatory process. (c) Obstructed olfactory cleft caused by anatomic anomalies and inflammatory process.

Patients who had complained of olfactory dysfunction were asked about their medical histories and examined by nasal endoscopy and a sinus CT. The olfactory function was evaluated by a phenyl ethyl alcohol (PEA) threshold test. Those who were suspected to be suffering from sensorineural olfactory loss, such as a history of loss of olfactory function during childhood or after an accident involving head trauma or an episode of upper respiratory infection, were excluded. Those whose nasal endoscopy revealed mucopurulent discharge, polyps, or tumor masses in the nasal cavities, including the OC, or scar tissue in the OC due to a prior sinus surgery were also excluded from the study. When the CT uncovered that the olfactory cleft was obstructed, but the sinuses were clear or the disease severity of the sinusitis was mild, they were diagnosed with obstructed OC disease based on Biacabe et al.'s definition [2] and enrolled in the study. The etiology of olfactory cleft obstruction is classified into anatomic deformity, inflammatory process, or both based upon the CT findings (Figures 1(a)–1(c)).

Eligible patients were medically treated over a 2–4 week-long dosage using oral corticosteroids (10 mg prednisolone twice per day), followed by a 3-month low-dose erythromycin (250 mg twice per day) with intranasal corticosteroids (beclomethasone dipropionate, 2 puffs twice per day). The low-dose erythromycin has been assumed to have anti-inflammatory and immune-modulatory effects in chronic sinonasal diseases [9]. If the patient's olfactory function did not improve, or the improvement of olfactory function was not deemed satisfactory by the patient, use of an endoscopic olfactory cleft opening was advised to open the obstructed olfactory cleft. This study was approved by the Ethics Committee of Taichung Veterans General Hospital (IRB TCVGH no.: CE18198 A). The authors assert that all procedures contributing to this work comply with the ethical standards of the relevant national and institutional guidelines on human experimentation of Taichung Veterans General Hospital and with the Helsinki Declaration of 1975, as revised in 2008.

### 2.2. Endoscopic Olfactory Cleft Opening

**Figure 2:**
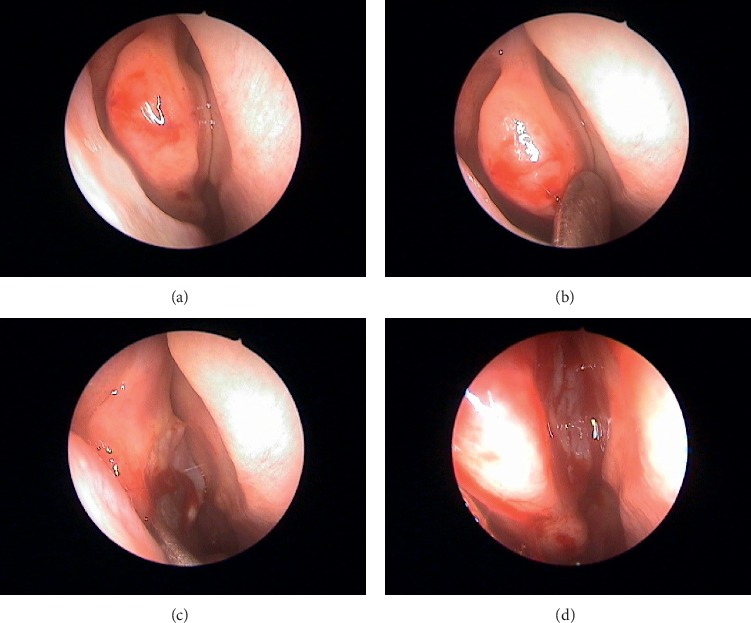
(a) Preoperative endoscopic picture. (b) The right middle turbinate lateralized using an elevator. (c) The right olfactory cleft opened. (d) Edematous mucosa seen in the right olfactory cleft.

The endoscopic OC opening was performed under local anesthesia. The patient was first placed in the supine position, and after receiving a topical anesthesia, a 2% xylocaine/epinephrine solution was injected into the middle turbinate. Under an endoscopy, the middle and superior turbinates were gently lateralized to open the olfactory cleft using an elevator (Figures 2(a)–2(d)). The middle and superior turbinates were lateralized as much as possible to touch the lateral nasal wall. After the middle and superior turbinates were lateralized, the OC was inspected. If polypoid masses were observed inside the OC, a biopsy was done to rule out the possibility of tumor, such as respiratory epithelial adenomatoid hamartoma. Finally, a piece of gelform was placed in the OC. Patients were treated with intranasal corticosteroids after surgery. Any adverse effect during the operation and every subsequent postoperative visit was recorded.

### 2.3. PEA Threshold Test

The PEA threshold test was used to evaluate each patients' olfactory function preoperatively and during follow-up after surgery. A two-alternative, forced-choice single-staircase paradigm was employed for the PEA threshold test. It consisted of the presentation of two glass bottles for each subject. One bottle contained a PEA odorant, whereas the other was a blank control. The two bottles were individually presented beneath the subject's nose in a random order. The subject then indicated which one possessed the stronger odor. If no difference was perceived by the subject, a guess was required. The test began with one of the pair of bottles containing a PEA odorant level at 10^−6^ log vol/vol and PEA concentrations which ranged from 10^−1^ to 10^−9^ log vol/vol, in half-log concentration steps. Correct identification of the bottle which contained the PEA odorant in five successive trials triggered a reversal of the staircase to the next lower concentration, whereas a single incorrect identification triggered the reversal of the staircase to the next higher concentration. Subsequently, correct identification of the bottle that contained the PEA odorant in two successive trials triggered a reversal of the staircase to the next lower concentration. When a total of seven reversals were acquired, the test was completed. The geometric mean of the last four reversed points was used as a threshold estimate.

### 2.4. Statistical Analysis

Patients who received endoscopic OC opening were divided according to the history of prior sinus surgery and the etiology of olfactory cleft obstruction. The effect of endoscopic OC opening on olfaction was compared between different groups. All data are presented as mean ± standard deviation (SD). The PEA thresholds were compared both before and after endoscopic OC opening through use of the Wilcoxon signed rank test. The improvement rate in the olfactory thresholds was compared between patients who had not undergone a prior sinus surgery with those who had had prior sinus surgery by Pearson's chi-square test. Results were compared amongst the 3 groups of etiology by Fisher's exact test. The postopening decrease of the PEA threshold was compared between patients who did not receive any prior sinus surgery with those who had had prior sinus surgery by use of the Mann–Whitney *U* test. The postopening decrease of the PEA threshold was compared amongst the 3 groups of etiology by the Kruskal–Wallis test, and a post hoc test was performed using the Scheffe test. All computations were performed using SPSS version 17.0 (SPSS, Inc., Chicago, IL, USA). Two-tailed *p* values <0.05 were considered statistically significant.

## 3. Results

### 3.1. Demographic Data

Forty-two patients with obstructed OC disease who underwent bilateral endoscopic OC opening procedures were analysed in this study. There were 19 male and 23 female patients, with their ages ranging from 23 to 70 years, with a mean of 48.9 years (SD = 11.62). Among them, 16 patients had undergone prior sinus surgery for rhinosinusitis and 18 complained of nasal obstruction. After receiving endoscopic OC opening, only 5 patients still complained of nasal obstruction.

The olfactory cleft was obstructed only by anatomic anomalies (A group) in 14 patients, only by inflammatory process (I group) in 14 patients, and by both anatomic anomalies and inflammatory process (A and I group) in 14 patients. In patients whose OCs were obstructed by inflammatory process, edematous or polypoid mucosa was observed inside the OC. Mucus stagnation was also seen inside the OC in some patients. In patients whose OCs were obstructed only by anatomic anomalies, the OC is nearly free of any lesion. The anatomic anomalies included a deviated nasal septum, concha bullosa, and paradoxical turbinates. There were 5 patients with concha bullosa, 1 patient with deviated nasal septum, 3 patients with concha bullosa and deviated nasal septum, 2 patients with paradoxical middle turbinate, 2 patients with paradoxical superior turbinate, and 1 patent with concha bullosa and paradoxical superior turbinate in A group, and there were 5 patients with concha bullosa, 4 patient with deviated nasal septum, 3 patients with concha bullosa and deviated nasal septum, and 2 patients with paradoxical middle turbinate in the A and I group.

### 3.2. Postoperative Change of Olfactory Function

The preoperative and postoperative PEA thresholds are shown in Table 1. The PEA threshold ranged from −1 to −5.625 with a mean of −1.5268 before surgery and ranged from −1 to −9 with a mean of −4.0476 after surgery. The PEA threshold decreased significantly after surgery (*p* < 0.001). The olfactory threshold decreased postoperatively in 32 patients. The improvement rate was 76.2%. Postoperative follow-up ranged from one to 33 months with a mean of 8.5 months.

### 3.3. Comparison of Postoperative Change of Olfactory Function between Patients with and without Prior Sinus Surgery

When patients were divided according to any history of previous sinus surgery, the olfactory threshold decreased after surgery in 18 of the 26 patients who did not have prior sinus surgery and in 14 of 16 patients who had undergone prior sinus surgery. The improvement rate was not significantly different between the 2 groups (*p*=0.27). The mean PEA threshold decreased significantly after surgery in both groups (Table 1), but the change in the PEA threshold was not significantly different between the 2 groups (*p*=0.677). Postoperative follow-up ranged from one to 33 months with a mean of 10 months in those who did not have prior sinus surgery and from one to 23 months with a mean of 5.9 months in those who had undergone prior sinus surgery.

### 3.4. Comparison of Postoperative Change of Olfactory Function in Patients with Different Etiologies of Obstruction

When the patients were classified by their etiology of obstruction, the olfactory threshold decreased after surgery in 7 of the 14 patients in the A group, in 13 of the 14 patients in the I group, and in 12 of the 14 patients in the A and I group. The improvement rate was significantly higher in the I group than in the A group (*p* = 0.033). The mean PEA threshold decreased significantly after surgery in all 3 groups (Table 1), but the change in the mean PEA threshold was not significantly different between the 3 groups (*p* = 0.091). Postoperative follow-up ranged from one to 26 months with a mean of 8.9 months in the A group, from one to 21 months with a mean of 7.3 months in the I group, and from one to 33 months with a mean of 9.2 months in the A and I group.

### 3.5. Operative Complication

The most common operative complication was headache. Fourteen patients complained of headache during surgery; however, their headache abated quickly after surgery. No episode of epistaxis, rhinosinusitis, or cerebrospinal fluid rhinorrhea occurred in any patient during postoperative follow-up.

## 4. Discussion

The OC is the most important anatomic location for maintaining olfactory function [10]. Obstruction of the OC will therefore obviously impair olfactory function [3]. The OC may become obstructed due to different etiologies, such as anatomic anomalies, inflammatory process, polyps, or tumors [2, 6, 7, 11].

When the OC is obstructed by polyps or tumors, the endoscopic removal of the polyps or tumors has been reported to restore olfactory function [7, 11]. On the contrary, the local mucosal inflammation in the OC is often not responsive to the use of corticosteroids and antibiotics, partly because the OC is a narrow passage and/or the mechanism of olfactory dysfunction in inflammatory sinonasal diseases may include both transport and sensory factors [1, 2]. Furthermore, if the OC is obstructed by anatomic anomalies, medical treatment has also been shown to be ineffective in restoring olfactory function [2].

In this study, we have excluded patients with OCs obstructed by polyps or tumors and included only those with OCs obstructed by anatomic anomalies and/or inflammatory process. Our results show that when the OC is obstructed by mucosal inflammation with/without mucus stagnation, endoscopic opening of the OC was a better option for improving olfactory function than was medical treatment. The olfactory threshold improved in 13 of 14 patients whose OCs were obstructed only by inflammatory process. In contrast, Biacabe et al. [2] reported that the olfactory threshold improved in 3 of 6 patients whose OCs were obstructed only by inflammatory process through the use of medical treatment.

We assume that the opening of the OC may improve the ventilation and draining of the OC, which in turn helps reduce local inflammation in the OC, while also providing easier access for both odorants and topical corticosteroids to reach the olfactory epithelium. However, when the olfactory function did not return to normal in most patients, sensory olfactory dysfunction might have been associated in these patients. The release of mediators from any inflammatory cells in an obstructed OC has been suspected as a cause of damage to the olfactory epithelial cells, resulting in sensorineural olfactory dysfunction [10].

Conversely, when the OC was obstructed only by anatomic anomalies, the patient's improvement rate was significantly lower in this study. When Biacabe et al. [2] treated patients whose OCs were obstructed only by anatomic anomalies using corticosteroids, the olfactory function did not improve in any patient. Jankowski et al. [8] treated 3 patients with constitutional olfactory cleft stenosis by OC dilatation. Improvement in dysosmia was achieved after surgery. However, an inflammatory process was also seen in the OC in all 3 cases. In contrast, our results show that surgical opening of the OC did not offer a result in patients with olfactory clefts obstructed only by anatomic anomalies as good as those with olfactory clefts obstructed only by inflammatory process. This indicates that the anatomic anomalies may need to be corrected through the use of septoplasty and/or the removal of concha bullosa.

The olfactory outcomes after opening of the OC were not different between patients who had or had not undergone a prior sinus surgery. This may be due to the fact that we had excluded patients with severe OC scarring which had been caused by a prior sinus surgery. Scar tissue in the OC is very difficult to manage. Biacabe et al. [2] treated a patient with scar tissue in his inferior olfactory cleft by administering corticosteroids, but his olfactory function did not improve.

A paradoxical curvature of the middle turbinate has been suggested as a possible predisposing factor for chronic rhinosinusitis [12], while a lateralized middle turbinate has also been considered to be one of the common causes of FESS failure [13]. Therefore, lateralization of the middle and superior turbinates through the use of endoscopic OC opening may both influence the patency of the ostiomeatal complex and disturb the mucociliary clearance in the ostiomeatal complex. However, no episode of rhinosinusitis had been observed in our patients after performing endoscopic OC opening.

It is also possible to injure the cribriform plate when the middle and superior turbinates are lateralized by an instrument. Although several patients complained of headache when their middle and superior turbinates were lateralized, and the headache was transient and no cerebrospinal fluid leakage occurred in any of those patients. It must be emphasized that the middle and superior turbinates should be gently lateralized.

There were several limitations in this study. The first was that the number of patients in each group was too small to reach a solid conclusion. The second was that the postoperative follow-up was short. The long-term effect needs further observation. The last was that postoperative CT was not performed in every case. The postoperative CT might explain why olfactory function improved in patients with inflammatory process instead of anatomic anomalies.

## 5. Conclusions

Our results show that endoscopic OC opening is both effective and safe in treating olfactory dysfunction in patients with OC disease caused by inflammatory process. It still remains unclear as to the role of endoscopic OC opening on OC disease caused by anatomic anomalies.

## Figures and Tables

**Table 1:** Comparison of preoperative and postoperative olfactory function.

	Preoperative	Postoperative	*P* value
Mean PEA threshold (42^*∗*^)	−1.5268 ± 1.05623	−4.0476 ± 2.82096	<0.0001
Sinus surgery history			
Without a prior sinus surgery (26) mean PEA threshold	−1.5962 ± 0.96586	−4.0625 ± 3.04205	<0.0001
With a prior sinus surgery (16) mean PEA threshold	−1.4141 ± 1.22875	−4.0234 ± 2.56751	0.001
Etiology of olfactory cleft obstruction			
Anatomic anomaly (14) mean PEA threshold	−1.2768 ± 0.69021	−2.7411 ± 2.41811	0.050
Inflammatory process (14) mean PEA threshold	−1.8036 ± 1.45915	−4.8929 ± 3.01792	0.001
Anatomic anomaly and inflammatory process (14) mean PEA threshold	−1.5 ± 0.89738	−4.5089 ± 2.75893	0.002

^*∗*^Number of patients.

## Data Availability

The data used to support the findings of this study are available from the first author upon request.
